# Termination of Ventricular Tachycardia by Prepotential Exit Block

**DOI:** 10.19102/icrm.2026.17034

**Published:** 2026-03-15

**Authors:** Ozcan Ozeke, Dursun Aras, Serkan Topaloglu

**Affiliations:** 1 Department of Cardiology, University of Health Sciences, Ankara Bilkent City Hospital, Ankara, Turkey; 2 Department of Cardiology, İstanbul Medipol University, İstanbul, Turkey

**Keywords:** Discrete prepotential, exit block, isolated prepotential, prepotential, ventricular tachycardia

## Abstract

Discrete prepotentials (PPs) mapped within the aortic sinuses of Valsalva are considered reliable targets for the ablation of idiopathic premature ventricular complexes (PVCs) and ventricular tachycardias. A critical conduction time is required for the PVC to manifest, as evidenced by a distinct isoelectric segment between the discrete PPs and the ventricular electrograms, consistent with the limitations imposed by the local refractoriness of the tissue at the exit site.

## Case presentation

**Figure 1:**
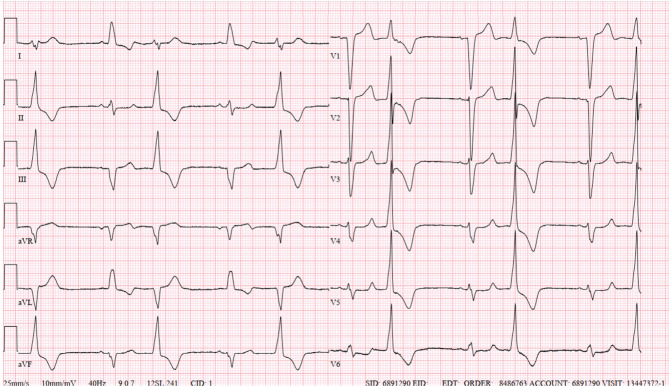
This 12-lead electrocardiogram shows a ventricular bigeminy with right bundle branch block morphology.

**Figure 2:**
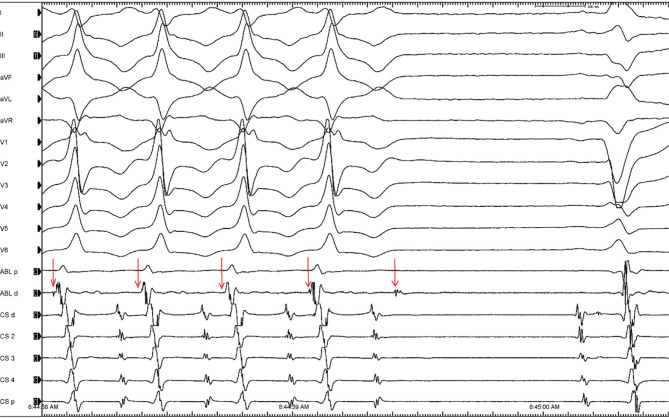
Termination of ventricular tachycardia by prepotential-to-myocardial exit block.

A 68-year-old man with left bundle branch block (LBBB) and reduced left ventricular (LV) systolic function (ejection fraction, 35%) was noted to have frequent premature ventricular contractions (PVCs) in patterns of bigeminy. Coronary angiography excluded coronary artery disease. The PVC morphology is shown in **Figure 1**, which had all the characteristics of PVCs arising from the LV summit. To improve cardiomyopathy, the patient was offered staged interventions, with ablation of PVCs as the first step, followed by cardiac resynchronization therapy (CRT). Upon arrival in the electrophysiology laboratory, the patient was noted to be in ventricular tachycardia (VT) **(Figure 2)**. Three-dimensional electroanatomic activation mapping during the PVCs was performed from the aortic sinuses of Valsalva and the LV outflow tract just beneath the commissure between the right and left sinuses of Valsalva of the aortic valve. At that site in the left sinus of Valsalva, a consistent prepotential (PP) was recorded during sinus rhythm with the ablation catheter **(Figure 2)**. However, this signal was likely buried within the QRS complex due to delayed LV activation secondary to LBBB.

## Discussion

This tracing illustrates the classic teaching: “*If a baseline ECG shows persistent bundle branch block, then the presence of the contralateral bundle branch block morphology during a wide complex tachycardia suggests VT.*”^1^ Moreover, it revealed that the rare phenomenon of VT termination was due to PP-to-myocardial exit block **(Figure 2)**. There has been a long-standing discussion about the mechanisms underlying repetitive ventricular ectopy: automaticity versus re-entry related to slow conduction.^1–8^ Some authors propose that PVC mechanisms likely differ between structurally normal (triggered activity or automaticity) and diseased hearts (micro–re-entry involving slow conduction).^1,5,8^ In this case, while the PVCs were likely due to triggered activity or abnormal automaticity **(Figure 1)**, slow conduction in scarred myocardium may also have played a role. A critical conduction time was required for the PVC to manifest, as evidenced by a distinct isoelectric segment between the discrete PPs and the ventricular electrograms, consistent with the limitations imposed by the local refractoriness of the tissue at the exit site.^1,9,10^ The stimulus–QRS complex latency could also be explained by the capture and exit of the tract from the arrhythmogenic origin, followed by depolarization of the myocardium with a conduction delay.^9^ Discrete PPs mapped inside the aortic sinuses of Valsalva are deemed reliable targets for the ablation of PVCs,^11–13^ as demonstrated in this case: PP-to-myocardial block resulted in VT termination, and ablation at this site led to complete elimination of the PVCs/VT. The patient’s ejection fraction improved only marginally after PVC ablation even though there was a complete abolition of PVCs, and he subsequently underwent successful CRT with the placement of a biventricular pacemaker.
